# General practice experiences for parents of children with intellectual disability: a systematic review

**DOI:** 10.3399/BJGPO.2023.0010

**Published:** 2023-06-28

**Authors:** Nicky Thomas, Helen Atherton, Jeremy Dale, Kayla Smith, Hayley Crawford

**Affiliations:** 1 Division of Health Sciences, Warwick Medical School, University of Warwick, Coventry, UK

**Keywords:** general practice, parents, informal carers, intellectual disability, experiences, caregivers

## Abstract

**Background:**

Parents of children diagnosed with intellectual disability are at increased risk of mental and physical health difficulties compared with other parents. They are likely to regularly seek medical treatment for their health concerns from general practice as well as on behalf of their child with intellectual disability, yet there is limited evaluation of the role general practice plays for this patient group.

**Aim:**

To explore parents’ experiences of general practice support when caring for a child with intellectual disability.

**Design & setting:**

Systematic review of studies reporting experiences of general practice as described by parents who care for children with intellectual disability.

**Method:**

Databases were searched using a pre-defined search strategy. Studies were included based on detailed inclusion criteria, title, abstract, and full-text screening. Quality assessment was conducted using the Mixed Methods Appraisal Tool (MMAT). A narrative synthesis was conducted.

**Results:**

A total of nine studies were identified. There was a clear absence of data on parents' own health experience and consultation in general practice. Findings related to navigating general practice on behalf of their child’s health including accessibility of general practice and positive and negative experiences of GPs.

**Conclusion:**

Findings from this review highlight priority areas for research, including further exploration of parents’ perspectives on seeking support specifically for their own health concerns, while caring for a child with intellectual disability, to bring more awareness and understanding of the role general practice plays in supporting the health of this carer group. This review also considers implications for clinical services, including tailoring appointments for this patient group as a priority for continuity of care, which may result in improved experiences of general practice and encourage better communication.

## How this fits in

This review synthesises existing literature and reports on experiences of using general practice from the perspectives of this informal carer group. There is very little evidence about the role general practice plays in supporting parents for their own health needs while caring for a child with intellectual disability. It does, however, provide further understanding of barriers and facilitators when seeking support on behalf of their child, and highlights a gap for studies exploring how this patient group experiences general practice to support their own health concerns.

## Introduction

Parents of children with intellectual disability are at higher risk of mental and physical health difficulties and overall poorer wellbeing over time compared with other parents.^1,2^ This continues for older parents of adult children and includes loss of identity owing to the continuous caring role.^3^ Furthermore, a 10-fold increase in anxiety and depression has been reported for this carer group since the COVID-19 pandemic,^4^ which is owing to increased care demands alongside reduced service support.^5,6^ However, other factors, such as genetic diagnosis, child age, and behavioural characteristics, can also indirectly influence parental wellbeing.^7^

General practice is an important pathway of health care for carers and the people they care for.^8^ However, little is known about the use of general practice or experiences of parents who are carers of children with intellectual disability when seeking mental or physical health support via general practice. While there is some evidence suggesting that parents of children with intellectual disability are less likely to visit their GP owing to caring demands,^9^ evidence suggests this patient group is more likely to seek medical treatment from general practice than parents of typically developing children.^10^ Additionally, previous research has indicated that this patient group is often in frequent contact with general practice for their own health, or for that of their child.^11^ In the UK, GPs provide incentivised annual health checks including a review of physical and mental health needs, coordination with secondary services, and appropriate referrals to reduce healthcare inequalities for people with intellectual disability.^12^ Hence, GPs are well-placed to stay in contact with this patient group and identify carers at risk of poor health to ensure appropriate referrals are made to sustain mental and physical wellbeing^13^ and offer relatable information.^14^

A previous systematic review suggested supportive interventions for carers provided by general practice consistently produced positive benefits on caregiver burden and depressive symptoms; however, this was specifically for informal carers of patients with dementia.^15^ A review focusing on parents of children with intellectual disability will increase understanding of caregiver satisfaction within general practice adding to the literature on carer support. This is of particular importance given that chronic stress, which is associated with parent carers, is higher than other carer groups such as dementia,^16^ and chronic stress can lead to increased mental and physical health difficulties.^9,17^ There is no systematic review, to the authors' knowledge, on experiences or perceptions of general practice as described by parents of individuals with intellectual disability. This review addresses this gap by synthesising literature on parents’ experiences when interacting with general practice for their own health concerns or for that of their child with intellectual disability, to provide further understanding of the role general practice plays for this carer group.

## Method

### Inclusion and exclusion criteria

The Preferred Reporting Items for Systematic reviews and Meta-Analyses (PRISMA) guidelines were followed for this review.^18^ This systematic review was conducted in accordance with the protocol (CRD42021298044) published in the international database of prospectively registered systematic reviews in health and social care (PROSPERO).

The inclusion criteria for the review were any study design and studies that investigated experiences or perspectives of parents who care for individuals, of any age, with an intellectual disability while interacting with general practice (or the equivalent in non-UK studies). Interaction in this context was defined as engagement with or use of general practice when seeking support for health concerns for themselves or their child; general practice included general practice staff, such as GP, general practice nurse, or administration, via any form of communication (for example, face-to-face consultation, telephone, or digital app). The inclusion criteria were also studies that included experiences of general practice concerning this parent carer group’s mental health, physical health, or wellbeing, or that of the individual they care for; studies that explored parent carers expressed unmet needs when utilising general practice; and studies published in English with no date restrictions. Studies were excluded where experiences could not be directly attributed to use of general practices services; for example, examining experiences across settings.

### Search strategy

Databases were searched in January 2022 and included MEDLINE (OvidSP), Cumulated Index to Nursing and Allied Health Literature (CINAHL), PsychINFO, Web of Science, and Scopus. Search terms included all relevant terms for the aims of the review. Reference sections of all included studies were also screened to identify any other eligible studies, and articles citing included studies were searched. An example of the full search strategy can be found in Supplementary Table S1.

### Screening and selection of studies

All duplicates were removed. Remaining title, abstracts, and full-text articles were independently screened by two reviewers against the inclusion criteria using Covidence software (version 2022). Studies that met the inclusion criteria at full-text stage were included in the review. Discrepancies were resolved by discussions between two reviewers. A third reviewer was consulted where a consensus could not be reached. Relevant data were extracted and recorded on a pre-defined data extraction form by two reviewers.

### Quality assessment

The MMAT (version 2018)^19^ was used to conduct quality assessment of all studies fitting the inclusion criteria. An overall quality rating was determined for contextual information only and studies were not excluded on this basis. Discrepancies were discussed, and a consensus was reached to give overall scores for each study.

### Data analysis

A narrative synthesis approach, which involves synthesising findings from included studies and using text and tables to summarise overall findings, was utilised.^20^ An iterative approach was followed, and initial description of results built a preliminary synthesis of studies identifying experiences highlighted by parents of general practice. Emerging patterns were explored within and between studies to understand why particular barriers or facilitators experienced affected the use of general practice and the role parents perceived it to play in supporting the health concerns of themselves or their child.

## Results

A total of 1755 results were screened, resulting in nine studies being included;^11,21–28^ see the PRISMA flowchart in Supplementary Figure S1, including reasons for exclusions.

The studies were published from 1998–2020 in the UK,^21,23,27,28^ Australia,^24–26^ Norway,^11^ and Republic of Ireland.^22^ Eight of the studies included both mothers and fathers, with one including mothers only.^22^ Characteristics of the children they cared for varied, with level of intellectual disability specified in only four studies.^22–28,28^ Children’s age ranged from 2–67 years, with one study not specifying age range.^27^ Of the nine included studies, five were qualitative,^11,21–26,26^ three quantitative,^24,25,27^ and one mixed method.^28^ All included studies focused on the experiences or perspectives of parents utilising general practice on behalf of their child with intellectual disability, with none of the studies reporting on general practice use for parents’ own health concerns. One study suggested the GP was used for parent carers’ health concerns but did not explore their experiences when seeking support for own health needs.^11^ Further study characteristics can be viewed in Supplementary Table S2.

### Quality assessment

Seven of the studies were rated high quality,^11,21–25–27–27^ and two were rated low quality,^24,28^ as three out of five of the study design criteria was not met according to the MMAT guide. Quality assessment ratings for each included study can be found in Supplementary Table S3.

### Reasons for use of general practice

All nine studies highlighted parent health service-related experiences and/or perspectives of utilising general practice for the health of their child with intellectual disability, including reasons for use.^11,21–28^ The most frequently expressed need described in five of the studies^11,22,25,26,28^ was for a supportive GP who understood the health complexities of individuals with intellectual disability, which often go beyond somatic or medical concerns (for example, communication difficulties and mental health problems). Five studies^21,22,24,27,28^ reported parents utilising the service for general healthcare concerns for their child including somatic issues or queries, and two studies^11,22^ described medication or prescriptions as a reason for use. Two studies^23,25^ gave parent accounts of seeking support for more specialised health concerns including support for their child’s mental health or challenging behaviour with varying outcomes. Similarly, two studies^22,26^ reported parents using general practice for further information about their child’s condition or health concern.

### Positive experiences of general practice as described by parents

Five studies^11,22,25,26,28^ described parents as having positive experiences with general practice when they believed their GP to be more understanding and supportive of their child’s health concerns. Four studies^11,22,26,28^ described what parents felt created a good relationship with their GP, including a GP who met the holistic health needs of the child and parent, and an accessible GP who understood the issues associated with disabled children, rather than just somatic health concerns. Both Doyle^22^ and Newton and McGillivray^26^ suggested a good relationship with a GP resulted in satisfaction of care for their child and improved parental coping with their caring role. Similarly, four studies^21,26–28^ valued the importance of continuity of care, describing how parents would go to great lengths to find a GP whom they could build a good relationship with.

### Negative experiences of general practice as described by parents

Seven studies^11,21–28,26,28^ described negative experiences when utilising general practice for their child’s health concerns. Six studies^11,21–24,28^ described GPs’ lack of knowledge about their child’s intellectual disability and lack of acknowledgement or understanding around parents’ wider concerns as a barrier when receiving support for their child’s health. This included parents seeking advice or referrals from GPs for behavioural or mental health difficulties.^11,23^ Three studies^11,21,26^ alluded to parents expressing their wishes for general practices to be more involved in coordinating overall health care of their child and participate in collaborative group meetings. One study reported parents’ concerns regarding transition support for their child from general practice owing to its lack of prior engagement with integrated services resulting in negative perspectives of GPs.^21^

### Accessibility of general practice

Two studies^11,26^ described accessing GP services as a barrier for parents when seeking support on behalf of their child as there was not enough time to talk through complex health concerns. The parents within these studies suggested longer consultations were needed along with better flexibility to accommodate their children with intellectual disability.

## Discussion

### Summary

This review examined the experiences of general practice as described by parents seeking healthcare support on behalf of their child with intellectual disability or for parents’ own health concerns. While none of the studies reported parents’ describing experiences using general practice for their own health concerns, this systematic review provides insight into their experiences of navigating general practice on behalf of their child. The most frequent use of general practice was for general healthcare discussion on behalf of their child, and positive experiences of general practice centred around a supportive and understanding relationship with GPs and continuity of care. Conversely, negative experiences included the lack of knowledge around the wider health difficulties of patients with intellectual disability and access to general practice.

### Strengths and limitations

To the authors’ knowledge, this is the first review to evaluate experiences for parents of children with intellectual disability concerning general practice. The review includes a range of study designs and countries with similar healthcare systems to the UK. This provides a wide overview of the literature, and although experiences consisted of a mixture of positive and negative experiences throughout the countries, only Norway and one study completed in Australia focused specifically on general practice as a standalone service.^11,26^ Although comparing a range of countries that explore the role of general practice for this patient group can be problematic owing to country-specific differences, it is clear further exploration of general practice service is needed.

Studies were included if they reported experiences of interacting with general practice for either the parent carer or child with intellectual disability and could be attributed directly to their experience with general practice and not to their wider experiences of health care. This meant excluding studies where experiences related to the wider healthcare landscape including general practice, and while this meant findings were solely related to general practice as intended, it may have excluded relevant perspectives.

Not all included studies reported the child’s age and/or severity of intellectual disability. Therefore, conclusions cannot be drawn on how demographic factors influence a parent’s experiences of navigating general practice. Health needs of individuals with intellectual disability are heterogeneous, and receiving health care requires accommodations specific to their needs, including age and severity of disability.^29^

Transition from child to adult services is a crucial area of concern in provision of health services for those with intellectual disability.^30^ This review was not designed to explore studies focused on transition although it was mentioned as an area of concern in one of the included studies,^21^ which reflects its importance for parents, and the need for further research to understand the role of general practice during transition of care for individuals with intellectual disability.

### Comparison with existing literature

This review found no studies describing parents’ experiences of general practice for their own health concerns when caring for a child with intellectual disability. Previous research suggests this group of parents are in frequent use of general practice,^10^ yet there is a lack of research investigating the role of general practice in supporting parent carers’ health concerns. Further investigation is needed, particularly since the COVID-19 pandemic has resulted in families who care for individuals with intellectual disability reporting increased demands of continual care with little or remote service support.^31^ Research that explored experiences of parents who care for children with other long-term conditions and disabilities suggested GPs lacked the knowledge and understanding about their child’s long-term conditions and parent’s wider concerns for their child when using general practice, which are similar findings to the negative experiences identified in this review.^32,33^

### Implications for research and practice

This review has highlighted a clear research gap in the literature for studies exploring parents who care for children with intellectual disability and their experiences of utilising general practice. Further investigations that explore this patient group’s perspectives will bring an awareness and understanding of the role that general practice plays in supporting this carer group’s health concerns. This will not only increase the body of literature in this area, but also inform policy and practice in relation to supporting the mental and physical health of this patient group, to ensure timely and accessible support and sustain their caring role long term.

Future research, particularly in the UK, needs to consider focusing on evaluating general practice as a unique service and not part of wider services of health care, as it is the first point of access for 90% of all initial NHS contacts.^34^ This will ensure findings can be deduced from datasets that solely explore accounts of general practice so recommendations can be made for policy and practice. Included studies did not examine the experiences of parents from Black and minority ethnic (BME) communities and these groups should be a priority for future research. Individuals diagnosed with intellectual disability from BME communities face additional complex barriers when utilising primary care, including lack of health information accessed in community languages and negative attitudes by service providers, with carers having limited support from social networks.^35,36^ Exploring the experiences and perspectives of accessing general practice services for BME communities will provide further understanding of the health inequalities faced, and examples of good and bad practice for recommendations to be made in practice.

Tailoring appointments for children with intellectual disability and their parents so that they are a priority for continuity of care may result in better experiences of using general practice for this patient group, and encourage better communication and satisfaction of care. Additionally, ensuring annual health checks are offered to the child and tailored to include parents’ health may be a welcome opportunity to facilitate support for parents’ health in general practice.

This review has highlighted specific barriers as described by parents seeking support on behalf of their child with intellectual disability. Children with intellectual disability are often a complex patient group, which may impact how much they utilise general practice for wider health concerns. Explorative studies with general practice staff, such as GPs, would be useful to gain further understanding of professionals' insights into parent carer expectations during consultations, and views on how parents and individuals with intellectual disabilities health needs are individually met.

## References

[1] Panicker AS, Ramesh S (2019). Psychological status and coping styles of caregivers of individuals with intellectual disability and psychiatric illness. J Appl Res Intellect Disabil.

[2] Patton KA, Ware R, McPherson L (2018). Parent-related stress of male and female carers of adolescents with intellectual disabilities and carers of children within the general population: a cross-sectional comparison. J Appl Res Intellect Disabil.

[3] Pryce L, Tweed A, Hilton A, Priest HM (2017). Tolerating uncertainty: perceptions of the future for ageing parent carers and their adult children with intellectual disabilities. J Appl Res Intellect Disabil.

[4] Willner P, Rose J, Stenfert Kroese B (2020). Effect of the COVID-19 pandemic on the mental health of carers of people with intellectual disabilities. J Appl Res Intellect Disabil.

[5] Alexander R, Ravi A, Barclay H (2020). Guidance for the treatment and management of COVID-19 among people with intellectual disabilities. J Policy Pract Intellect Disabil.

[6] Bourquin P, Delestre I, Joyce R (2020). The effects of coronavirus on household finances and financial distress. https://ifs.org.uk/publications/effects-coronavirus-household-finances-and-financial-distress.

[7] Baker K, Devine RT, Ng-Cordell E (2021). Childhood intellectual disability and parents’ mental health: integrating social, psychological and genetic influences. Br J Psychiatry.

[8] Gilson K-M, Davis E, Corr L (2018). Enhancing support for the mental wellbeing of parents of children with a disability: developing a resource based on the perspectives of parents and professionals. J Intellect Dev Disabil.

[9] Seymour M, Giallo R, Wood CE (2017). The psychological and physical health of fathers of children with autism spectrum disorder compared to fathers of children with long-term disabilities and fathers of children without disabilities. Res Dev Disabil.

[10] Gallagher S, Whiteley J (2013). The association between stress and physical health in parents caring for children with intellectual disabilities is moderated by children’s challenging behaviours. J Health Psychol.

[11] Fredheim T, Lien L, Danbolt LJ (2011). Experiences with general practitioners described by families of children with intellectual disabilities and challenging behaviour: a qualitative study. BMJ Open.

[12] NHS Employers (2009). Clinical directed enhanced services (DESs) for GMS contract 2008/9.

[13] Royal College of General Practitioners (2017). Mental health in primary care. https://www.rcgp.org.uk/representing-you/policy-areas/mental-health-in-primary-care.

[14] National Institute for Health and Care Excellence (2018). Learning disabilities and behaviour that challenges: service design and delivery. NG93. https://www.nice.org.uk/guidance/ng93.

[15] Greenwood N, Pelone F, Hassenkamp A-M (2016). General practice based Psychosocial interventions for supporting Carers of people with dementia or stroke: a systematic review. BMC Fam Pract.

[16] Totsika V, Hastings RP, Vagenas D (2017). Informal caregivers of people with an intellectual disability in England: health, quality of life and impact of caring. Health Soc Care Community.

[17] Griffin J (2021). Day by Day: Emotional Wellbeing in Parents of Disabled Children.

[18] Moher D, Liberati A, Tetzlaff J (2009). Preferred Reporting Items for Systematic Reviews and Meta-Analyses: the PRISMA statement. PLoS Med.

[19] Hong QN, Fàbregues S, Bartlett G (2018). The Mixed Methods Appraisal Tool (MMAT) version 2018 for information professionals and researchers. Education for Information.

[20] Popay J, Roberts H, Sowden A (2006). Guidance on the conduct of narrative synthesis in systematic reviews. A product from the ESRC methods programme. https://www.lancaster.ac.uk/media/lancaster-university/content-assets/documents/fhm/dhr/chir/NSsynthesisguidanceVersion1-April2006.pdf.

[21] Brown M, Higgins A, MacArthur J (2020). Transition from child to adult health services: a qualitative study of the views and experiences of families of young adults with intellectual disabilities. J Clin Nurs.

[22] Doyle C (2022). The importance of supportive relationships with general practitioners, hospitals and pharmacists for mothers who 'give medicines' to children with severe and profound intellectual disabilities. J Intellect Disabil.

[23] Faust H, Scior K (2008). Mental health problems in young people with intellectual disabilities: the impact on parents. J Appl Res Intellect Disabil.

[24] Foreman PJ, Neilands J (1991). Parental perceptions of services to children with intellectual disability. J Intellect Dev Disabil.

[25] Man J, Kangas M (2019). Service satisfaction and helpfulness ratings, mental health literacy and help seeking barriers of carers of individuals with dual disabilities. J Appl Res Intellect Disabil.

[26] Newton DC, McGillivray JA (2019). Perspectives of carers of people with intellectual disability accessing general practice: 'I'd travel to the ends of the earth for the right person'. J Intellect Dev Disabil.

[27] Stanley R, Ng J (1998). Primary health care provision for people with learning disabilities: a survey of parents. Journal of Learning Disabilities for Nursing, Health, and Social Care.

[28] Wilson C, Mansell I (2010). Access to health and social care services and information. Learning Disability Practice.

[29] Iezzoni LI, Agaronnik ND, Meeks LM, Neal-Boylan L (2020). Disability as Diversity: A Guidebook for Inclusion in Medicine, Nursing, and the Health Professions.

[30] Brown M, Macarthur J, Higgins A, Chouliara Z (2019). Transitions from child to adult health care for young people with intellectual disabilities: a systematic review. J Adv Nurs.

[31] Rose J, Willner P, Cooper V (2022). The effect on and experience of families with a member who has intellectual and developmental disabilities of the COVID-19 pandemic in the UK: developing an investigation. Int J Dev Disabil.

[32] Rapley T, May C, Smith N, Foster HE (2021). 'Snakes & ladders': factors influencing access to appropriate care for children and young people with suspected juvenile idiopathic arthritis—a qualitative study. Pediatr Rheumatol Online J.

[33] Satherley RM, Wolfe I, Lingam R (2021). Experiences of healthcare for mothers of children with ongoing illness, living in deprived neighbourhoods health and place. Health Place.

[34] NHS England (2013). Transforming primary care in London. https://www.england.nhs.uk/london/wp-content/uploads/sites/8/2013/11/Call-Action-ACCESSIBLE.pdf.

[35] Nocon A, Sayce L, Nadirshaw Z (2008). Health inequalities experienced by people with learning disabilities: problems and possibilities in primary care. Tizard Learning Disability Review.

[36] Emerson E, Robertson J (2002). Future demand for services for young adults with learning disabilities from South Asian and black communities in Birmingham.

